# Relationship between thyroid stimulating hormone and night shift work

**DOI:** 10.1186/s40557-016-0141-0

**Published:** 2016-10-06

**Authors:** So-Hyun Moon, Bum-Joon Lee, Seong-Jin Kim, Hwan-Cheol Kim

**Affiliations:** 1 Department of Occupational and Environmental Medicine, Inha University Hospital, Incheon, Republic of Korea; 2 Department of Social and Preventive Medicine, School of Medicine, Inha University, Incheon, Republic of Korea; 3 Department of Occupational and Environmental Medicine, School of Medicine, Inha University, Incheon, Republic of Korea

**Keywords:** Night shift, Thyroid stimulating hormone, Subclinical hypothyroidism

## Abstract

**Background:**

Night shift work has well-known adverse effects on health. However, few studies have investigated the relationship between thyroid diseases and night shift work. This study aimed to examine night shift workers and their changes in thyroid stimulating hormones (TSH) levels over time.

**Methods:**

Medical check-up data (2011–2015) were obtained from 967 female workers at a university hospital in Incheon, Korea. Data regarding TSH levels were extracted from the records, and 2015 was used as a reference point to determine night shift work status. The relationships between TSH levels and night shift work in each year were analyzed using the general linear model (GLM). The generalized estimating equation (GEE) was used to evaluate the repeated measurements over the 5-year period.

**Results:**

The GEE analysis revealed that from 2011 to 2015, night shift workers had TSH levels that were 0.303 mIU/L higher than the levels of non-night shift workers (95 % CI: 0.087–0.519 mIU/L, *p* = 0.006) after adjusting for age and department. When we used TSH levels of 4.5 ≥ mIU/L to identify subclinical hypothyroidism, night shift workers exhibited a 1.399 fold higher risk of subclinical hypothyroidism (95 % CI: 1.050–1.863, *p* = 0.022), compared to their non-night shift counterparts.

**Conclusions:**

This result of this study suggests that night shift workers may have an increased risk of thyroid diseases, compared to non-night shift workers.

## Background

Night shift work has well-known adverse effects on health. According to the Labor Standards Act, night shift work is defined as work that is performed between 10 PM and 6 AM the next day, and an estimated 10.2–14.5 % of wage workers in Korea (1.27–1.97 million people) perform night shift work [1]. Many studies have revealed that night shift work can cause cardiovascular diseases [2], sleep disorders [3], peptic ulcers [4], and breast cancer among women [5]. The effect of night shift work on health is mainly thought to be related to its interference with circadian rhythms [6]. For example, night shift work interferes with the worker’s natural circadian timing system, which disturbs their normal circadian rhythms and physiological functions [7]. Previous research has also revealed that night shift workers disturbed levels of prolactin [8], cortisol [9], and growth hormone [10].

Thyroid stimulating hormone (TSH) levels change in accordance with circadian rhythms, and with sleep patterns [11]. However, very little research has evaluated the association of thyroid diseases with night shift work. One study that examined thyrotropin rhythm and night shift workers [12], and another study examined a correlation between TSH levels and night shift work [13]. Nevertheless, few studies have examined TSH levels over time. Therefore, the present study aimed to evaluate the changes in TSH levels over time among female workers according to their night shift status.

## Methods

### Data collection

This study retrospectively evaluated data from employee medical check-ups that were performed at a university hospital in Incheon between 2011 and 2015. Our institutional review board approved the study’s retrospective design. Most night shift workers at the university hospital were nurses, therefore, we ultimately evaluated data from 967 female adult workers. The Employee medical check-ups were performed using the same requirements as general and special medical check-ups and workers were required to fast for at least 8 h. This study collected data regarding sex, age, height, weight, night shift work, and TSH levels. The normal ranges were defined as 0.17–4.5 mIU/L for TSH and 0.8–1.90 ng/dL free T_4_, which were analyzed using blood testing (Cobas E601; Roche Diagnostics, Manheim, Germany). Four workers with thyroid diseases (abnormal TSH and free T_4_ levels) were excluded from the final analyses. The workers were categorized according to age (≤29 years, 30–39 years, 40–49 years, and ≥50 years) and body mass index (BMI) (<25 kg/m^2^ or ≥25 kg/m^2^). Self-administered questionnaires were used to collect data regarding smoking, alcohol drinking, and exercise habits. Non-/ex-smokers were defined as individuals who had not smoked within the last 6 months, and current smokers were defined as individuals who had smoked within the last 6 months. Non-/moderate drinkers were defined as individuals who consumed ≤2 servings of alcohol per day and excessive drinkers were defined as individuals who consumed >2 servings of alcohol per day. Exercise status was categorized as regular exercise (at least 30/min of exercise once per week) or no regular exercise.

Night shift workers, (≥4 night shifts per month) were identified based on their status in 2015, when all night shift workers were required to undergo a special medical check-up Departmental status was defined as nursing (general ward, outpatient unit, emergency room, operating room, and/or intensive care unit) or other (e.g., support workers or administrative workers).

### Statistical analysis

The general linear model (GLM) was used to analyze annual differences in TSH levels between the night shift workers and non-night shift workers (2011 to 2015 The generalized estimating equation (GEE) was used to estimate the correlation among the repeated TSH measures throughout the 5-year period, with night shift work defined as the independent variable and TSH levels as the dependent variable. TSH levels were reported as a continuous variable for most analyses, and as a categorical variable for the odds ratio (OR) analyses (subclinical hypothyroidism: ≥4.5 mIU/L). Age and department were defined as confounding variables, and were included in the GEE analysis. All data were analyzed using SPSS software (version 19.0; SPSS Inc., Chicago, IL).

## Results

Characteristics of the 967 included workers, who were categorized as night shift workers (546 workers, 56.5 %) and non-night shift workers (421 workers, 43.5 %) are shown in Table 1. Night shift workers were significantly younger than non-night shift workers and more likely to be excessive drinkers. However, we did not observe any shift-specific differences in BMI, smoking status, and exercise status. We identified 654 nursing workers and 313 workers who were employed in other departments. Approximately 74.5 % of the nursing workers were night shift workers, while 80.7 % of the other workers were non-night shift workers.

**Table 1:** Characteristics of study subjects according to night shift work

		Night shift work					
		Total	No		Yes		
		n	n	%	n	%	*P*-value^a^
Total		967	421	43.5 %	546	56.5 %	
Age (years)	≤29	364	84	20.0 %	280	51.3 %	<0.001
	30–39	338	165	39.2 %	173	31.7 %	
	40–49	229	148	35.2 %	81	14.8 %	
	≥50	36	24	5.7 %	12	2.2 %	
Body mass index (kg/m^2^)	<25	883	381	90.5 %	502	91.9 %	0.490
	≥25	84	40	9.5 %	44	8.1 %	
Smoking	Non-/ex-smoker	966	420	100 %	546	100 %	0.059
	Current smoker	1	1	0.0 %	0	0.0 %	
Drinking	Non-/moderate drinker	683	328	77.9 %	355	65.0 %	<0.001
	Excessive drinker	284	93	22.1 %	191	35.0 %	
Regular exercise	No	421	71	16.9 %	76	13.9 %	0.120
	Yes	546	350	83.1 %	470	86.1 %	
Department	Nursing	654	167	39.8 %	487	89.0 %	<0.001
	Other	313	252	60.2 %	61	11.0 %	

^a^Calculated using the chi-square test

The annual mean TSH levels for each group are shown in Table 2. The average TSH levels were 3.27 mIU/mL among night shift workers and 2.98 mIU/mL among non-night shift workers; the difference was not statistically significant for each year.

**Table 2:** Annual average thyroid stimulating hormone levels according to night shift work status

	Year	Total	Night shift work						
			No			Yes			
		n	n	Mean	SD	n	mean	SD	*p*-value^a^
TSH	2011	553	305	2.60	2.49	248	2.84	2.06	0.194
	2012	602	322	2.41	2.11	280	2.63	1.82	0.149
	2013	776	360	2.73	1.68	416	2.93	1.96	0.058
	2014	831	367	2.68	2.21	464	2.89	2.21	0.120
	2015	967	421	2.98	2.16	546	3.27	2.80	0.106

*TSH* thyroid stimulating hormone, *SD* standard deviation

^a^Analyzed using analysis of covariance (adjusted for age)

TSH levels were treated as single points in the GLM analysis to evaluate whether night shift work was associated with a linear change in TSH levels. After adjusting for age, we observed that night shift work was not associated with changes in TSH levels at each year with night shift workers having higher TSH levels (Table 3).

**Table 3:** TSH level according to night shift work from 2011 to 2015ª

TSH	Year	n	ß^d^	95 % CI	*P*-value
GLM^b^	2011	553	0.245	−0.140–0.631	0.212
	2012	602	0.250	−0.088–0.589	0.148
	2013	776	0.278	−0.008–0.564	0.057
	2014	831	0.215	−0.064–0.566	0.118
	2015	967	0.284	−0.060–0.628	0.106
GEE^c^	2011-2015	967	0.303	0.087-0.519	0.006

^a^The models were adjusted for age (GLM) or age and department (GEE). TSH was a continuous variable

^b^The GLM was used to evaluate the relationship between night shift work and TSH levels from 2011 to 2015

^c^The GEE was used to evaluate the relationship between night shift work and TSH levels during the 5 years

^d^ß means difference between night shift workers and non- night shift works

As the GLM cannot evaluate repeated measures over an observed period, we performed a GEE analysis to evaluate the relationship between night shift work and TSH levels during 2011–2015 (Table 3). The GEE analysis included 967 workers, and included age and department as covariates. Night shift workers had TSH levels that were 0.303 mIU/L higher, compared to the non-night shift workers (*p* = 0.006).

We also performed GLM and GEE analyses to evaluate the risk of subclinical hypothyroidism (TSH levels of ≥4.5 mIU/L) (Table 4). In the GLM analysis, night shift workers had not risk of subclinical hypothyroidism, compared to non-night shift workers except in 2012 (OR : 1.912, *p* = 0.025). In the GEE analysis, night shift workers had a 1.399-fold higher risk of subclinical hypothyroidism, compared to non-night shift workers (*p* = 0.022) (Table 4).

**Table 4:** The association between night shift work and subclinical hypothyroidism

		OR	95 % CI	*P*-value
GLM^a^	2011	1.246	0.740–2.484	0.409
	2012	1.912	1.086–3.364	0.025
	2013	1.338	0.862–2.079	0.195
	2014	1.441	0.919–2.259	0.112
	2015	1.245	0.878–1.765	0.219
GEE^b^	2011–2015	1.399	1.050–1.863	0.022

^a^The models were adjusted for age with TSH of ≥4.5 mIU/L as a categorical variable (GLM: each year, GEE: over the 5-year period)

^b^Adjusted for age and department

## Discussion

Serum TSH measurement is the most sensitive method for identifying thyroid dysfunction [14] However, few studies have evaluated whether night shift workers develop thyroid diseases, and only a few studies have examined the relationship between TSH levels and night shift work. In a study suggesting TSH levels with sleep time [12] and another study shows that insomnia is associated with an increased risk of thyroid cancer among postmenopausal women [15]. Moreover, a cross-sectional study of male night shift workers revealed that night shift workers had significantly higher TSH levels, compared to their day shift counterparts [13].

In the present study, we retrospectively evaluated longitudinal data regarding TSH levels, and found that night shift workers exhibited higher TSH levels, compared to day shift workers, in the age-adjusted annual GLM analyses but not statistically significant.. However, the GEE analyses (adjusted for age and department) confirmed that the TSH levels were higher among night shift workers over the 5-year study period. The GEE analysis was adjusted for department because approximately 70 % of the nurses were night shift workers.

Our results suggest that night shift work might be associated with the risk of subclinical hypothyroidism, and that this risk increased with longer employment as a night shift worker. Subclinical hypothyroid is a condition with normal free T_4_ levels and elevated TSH levels [16], which is exclusively diagnosed using screening test results. In the general population, subclinical hypothyroidism has a reported prevalence of 4–15 %, which varies according to the specific study [17]. When anti-thyroid peroxidase autoantibodies are present, there is a 25–50 % risk of subclinical hypothyroidism progressing to overt hypothyroidism within 20 years. If autoantibodies are not present, TSH levels of >3.0–4.5 mIU/L are considered a risk factor for progression, and regular observation is recommended. The current guidelines for the initial treatment of subclinical hypothyroidism recommend starting drug treatment at TSH levels of >10 mIU/L (while considering other co-existing conditions), although it remains unclear whether drug treatment is beneficial for patients with TSH levels of 4.5–10 mIU/L [18–21].

In the present study, we found that age-adjusted TSH levels of ≥4.5 mIU/L among night shift workers during 2011–2015 were associated with a 1.4-fold higher risk of subclinical hypothyroidism, compared to non-night shift workers. In that analysis, we adjusted for age because TSH levels are known to increase with age [21].

There are several potential explanations for why TSH levels were higher among night shift workers, compared to non-night shift workers. First, TSH levels exhibit a normal circadian rhythm, with study-specific peaks at approximately 2–4 AM and troughs at approximately 4–8 PM [11]. However, this circadian rhythm assumes that workers have a normal sleep at night, and night shift work-related changes in sleep schedule, timing, and quality may alter the body’s normal circadian rhythm and lead to an abnormal TSH circadian rhythm. Furthermore, some authors have suggested that sleep deprivation promotes oscillations in the TSH circadian rhythm [22], which increases the likelihood that TSH levels rise when workers are deprived of sleep after their night shift.

Second, other studies have found that night shift work disturbs women’s circadian rhythm and induces changes in their female hormone levels [23], reproductive system [24], and menstrual cycle [25]. Thus, women might be more sensitive to night shift work-related hormonal changes that could alter TSH levels.

Third, nocturnal eating may affect hormone levels (e.g., TSH, insulin, and glucagon) [26], and it is possible that night shift work might lead to irregular eating habits and nocturnal eating, which might lead to increases in TSH levels.

Fourth, some studies have also found that night shift work can increase the risk of autoimmune disease and altered immune system function [27], which might lead to increased TSH levels among night shift workers.

Although the present study was not designed to identify the causal factors that lead to the increased TSH levels among night shift workers, we did observe an increase in TSH levels among night shift workers, compared to non-night shift workers. Furthermore, we assume that night shift work might increase the risk of subclinical hypothyroidism.

The present study has several limitations that warrant consideration. First, we only evaluated female workers at a hospital (as most workers were female and/or nurses), and it is possible that our results may not be observed among men. Second, there was noticeable heterogeneity in the amount and type of night shift work, which included traditional night shifts, day and night shifts, and on-call shifts. This heterogeneity may limit the validity of our analyses. Third, our data were obtained from employee medical check-ups, and it is possible that our data regarding special diseases or drug history might not be accurate. Fourth, we defined the workers’ departments and night shift work statuses based on their status in 2015, and it is possible that not all individuals in the night shift worker group were consistently working night shifts throughout the study period. Fifth, blood samples were obtained at different times, and it is possible that the hormone levels did not reflect circadian rhythm-specific changes, as night shift workers were evaluated during their night shift, while day shift workers were evaluated during their day shift.

Despite these limitations, this study used cross-sectional repeated measures data from a large sample during a 5-year period, which may help overcome these limitations.

## Conclusions

In conclusion, we found that night shift work was associated with increased TSH levels among female workers at an university hospital. We believe that these findings may help increase awareness of thyroid disease occurrence among night shift workers. Furthermore, we hope that this study can provide the foundation for detailed studies regarding the effects of night shift work on thyroid function and disease, and the association between night shift work duration and TSH levels.

## Abbreviations

Free T4: Free thyroxine
GEE: Generalized estimating equation
GLM: General linear model
TSH: Thyroid stimulating hormone
